# Racial, Ethnic, and Geographic Disparities in Immunization Rates Among Patients With Inflammatory Bowel Disease

**DOI:** 10.1093/crocol/otad078

**Published:** 2023-12-14

**Authors:** Poonam Beniwal-Patel, Gabrielle Waclawik, Keely Browning, Aijan Urmat, Trevor L Schell, Ryan Smith, Antonio Huerta, Lauren Hipp, Sonya Dave, Neemit Shah, Kayla E Dillon, Katelyn Reiter-Schreurs, Rachel K Russ, Miguel A Mailig, Fauzia Osman, Francis A Farraye, Jennifer Weiss, Mary S Hayney, Freddy Caldera

**Affiliations:** Medical College of Wisconsin, Division of Gastroenterology and Hepatology, Milwaukee, WI, USA; Department of Medicine, University of Wisconsin School of Medicine & Public Health, Madison, WI, USA; Medical College of Wisconsin, Division of Gastroenterology and Hepatology, Milwaukee, WI, USA; School of Pharmacy, University of Wisconsin-Madison, Madison, WI, USA; Department of Medicine, University of Wisconsin School of Medicine & Public Health, Madison, WI, USA; Department of Medicine, Division of Gastroenterology and Hepatology, University of Wisconsin School of Medicine & Public Health, Madison, WI, USA; University of Wisconsin School of Medicine & Public Health, Madison, WI, USA; Department of Medicine, Medical College of Wisconsin, Milwaukee, WI, USA; Department of Medicine, Medical College of Wisconsin, Milwaukee, WI, USA; Depatrment of Medicine, Medical College of Wisconsin School of Medicine, Milwaukee, WI, USA; School of Pharmacy, University of Wisconsin-Madison, Madison, WI, USA; School of Pharmacy, University of Wisconsin-Madison, Madison, WI, USA; School of Pharmacy, University of Wisconsin-Madison, Madison, WI, USA; School of Pharmacy, University of Wisconsin-Madison, Madison, WI, USA; Department of Medicine, University of Wisconsin School of Medicine & Public Health, Madison, WI, USA; Inflammatory Bowel Disease Center, Department of Gastroenterology and Hepatology, Mayo Clinic, Jacksonville, FL, USA; Department of Medicine, Division of Gastroenterology and Hepatology, University of Wisconsin School of Medicine & Public Health, Madison, WI, USA; School of Pharmacy, University of Wisconsin-Madison, Madison, WI, USA; Department of Medicine, Division of Gastroenterology and Hepatology, University of Wisconsin School of Medicine & Public Health, Madison, WI, USA

**Keywords:** inflammatory bowel diseases, influenza vaccine, pneumococcal vaccines, recombinant zoster vaccine

## Abstract

**Background and Aims:**

Racial and ethnic disparities exist in the treatment of IBD. These disparities exist in adult vaccine uptake among the general population and may extend to patients with IBD. The primary aim of this study was to determine whether racial, ethnic, or geographic disparities existed in influenza vaccine uptake among patients with IBD.

**Methods:**

We performed a multicenter, retrospective cohort study evaluating adult vaccine uptake among patients with IBD seen at two tertiary referral centers between September 2019 and February 2020. The primary outcome was to determine if racial/ethnic and geographic disparities existed in influenza vaccine uptake for the two prior seasons. Our secondary outcomes were to determine if disparities existed for pneumococcal, zoster, or hepatitis B vaccines.

**Results:**

Among the 2453 patients who met the inclusion criteria, most identified as non-Hispanic White (89.9%), were on immunosuppressive therapy (74.5%), and received the influenza vaccine in both seasons (56.0%). Older age (prevalence ratio (PR) 0.98; 95% confidence interval (95%CI) 0.98-0.99; *P* < .001) and non-Hispanic White patients (PR 0.76, 95%CI 0.59–0.98, *P* < 0.03) were significantly more likely to be immunized. Black patients (PR 1.37; 95%CI 1.18–1.59; *P* < .001) and those living in underserved geographic areas (PR 1.35; 95%CI 1.17–1.56; *P* < 0.001) were less likely to be immunized. Racial/ethnic and geographic disparities were identified for pneumococcal, zoster, and hepatitis B vaccine uptake.

**Conclusions:**

Racial and ethnic vaccination uptake disparities exist among patients with IBD; patients from medically underserved areas are also vulnerable to these disparities Studies identifying patient, provider, and system-level opportunities to address these disparities are needed.

## Graphical Abstract

**Graphical Abstract:**
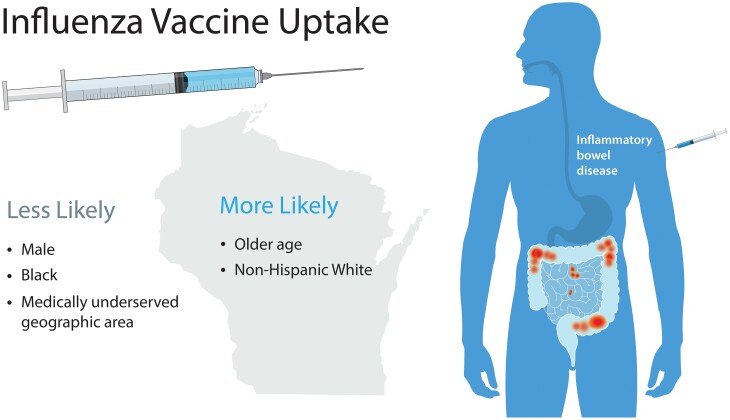

**Key messages**

**What is already known?**

Among patients with inflammatory bowel disease, studies have shown that racial disparities exist with access to subspecialty care, access to IBD therapy, and post-operative surgical outcomes.

**What is new here?**

Racial, ethnic, and geographic disparities exist in influenza, pneumococcal, and herpes zoster vaccine uptake compared to non-Hispanic Whites.

**How can this study help patient care?**

Racial and ethnic vaccination uptake disparities exist among patients with IBD; patients from medically underserved areas are also vulnerable to these disparities, highlighting the importance of considering geographic and patient-level factors when addressing vaccination uptake disparities.

## Introduction

Although all individuals should be current with immunization recommendations, patients with inflammatory bowel disease (IBD) have higher immunization rates compared to the general population but are still suboptimal with yearly influenza and pneumococcal vaccine uptake (48% and 75%, respectively).^1^ A recent study from the Centers for Disease Control and Prevention (CDC) showed that Black and Hispanic people account for at least 18% of all adults with an IBD diagnosis in the United States.^2,3^ This group has traditionally lagged in vaccine uptake.^3^ It is important that we better understand how this vital preventative service is being adopted within this medically and socially vulnerable population.

Geographic disparities in COVID-19 vaccine uptake in the general population as well as among patients with IBD, especially those who reside in a rural zip code or identify as an underrepresented minority have been identified.^4,5^ Racial and ethnic disparities in medical treatment and preventive health among patients with IBD have been documented, but vaccination disparities among patients with IBD have been understudied.^1,6^ The primary aim of this study was to determine if racial, ethnic, and geographic disparities exist in influenza vaccine uptake among patients with IBD. Our secondary outcomes were to determine whether disparities existed for other adult vaccines: pneumococcal, herpes zoster, or hepatitis B. We hypothesized that even though patients with IBD are at increased risk for VPD and vaccine uptake in this population has increased, the racial, ethnic, and geographic disparities seen in the general population would also exist in the IBD population. These groups likely have multifactorial reasons for low vaccine uptake that need to be further investigated.

## Methods

### Study Setting

We performed a multicenter, retrospective cohort study of patients with IBD receiving gastroenterology-specific ambulatory care from September 3, 2019 to February 28, 2020, at the University of Wisconsin Hospital (UW) or Medical College of Wisconsin Hospital (MCW).

### Study Population and Design

An EPIC system (EPIC Corporation) electronic health record (EHR) query was performed to identify patients who had ≥ 1 ICD-10 code (K50.xx for Crohn’s disease and K51.xx for Ulcerative colitis) during the study period.^7^ All charts were reviewed via manual abstraction to make sure they fit the following inclusion criteria: established diagnosis with IBD (Crohn’s disease, ulcerative colitis, and indeterminate colitis) and age ≥18 years seen in the gastroenterology clinic during the study period. Patients were excluded if they did not have race/ethnicity indicated in their medical record or an active Wisconsin Immunization Registry (WIR) record.

Sociodemographic characteristics, immunizations, and IBD-specific medication data were manually abstracted from the EHR. We defined immunosuppressive therapy as any of the following medications: azathioprine, 6-mercaptopurine, methotrexate, anti-tumor necrosis factor (anti-TNF), ustekinumab, tofacitinib, and systemic corticosteroid therapy (eg, prednisone). Non-immunosuppressive agents included: no IBD-directed therapy, aminosalicylate monotherapy, or vedolizumab monotherapy. Vedolizumab was considered in this group because previous studies have shown that it does not appear to impact vaccine response and is gut-selective.^8^ Sociodemographic classification included patient age, sex, race, ethnicity, and zip code of permanent residence at the time of data collection. Race was defined using the existing structure of the EHR data as self-identified by the patient, where White, Black, Asian, American Indian, Native Hawaiian/Pacific islander race are defined categorically, and Hispanic ethnicity is a modifier.^9^ Given the small sample size of certain racial and ethnic groups, patients were aggregated into two larger cohorts: non-Hispanic White patients and all racial and ethnic groups except non-Hispanic White patients for some analyses.

Using ZIP codes, the cohort was divided into urban (population > 10 000) or rural (population < 10 000).^10,11^ We excluded ZIP codes that are non-residential (eg, only P.O. Box, or commercial organization addresses), that correspond to populations of less than 500, or are located outside of Wisconsin. To further investigate disparities within rural and urban communities, the study cohort was divided into six geodisparity categories (rural underserved, rural, rural advantaged, urban underserved, urban, and urban advantaged) that incorporates information on regional healthcare capacity and health needs in Wisconsin ZIP Code Tabulation Areas (ZCTA) to create a comprehensive rural–urban geodisparity model. These Wisconsin ZCTAs and their corresponding categories can be downloaded at https://www.hipxchange.org/RuralUrbanGroups. The urban–rural, advantaged-underserved categories were determined using rates of poverty, uninsured, Medicaid, educational attainment, access to health care providers, and health status.^10,11^

### Wisconsin Immunization Registry

Patient immunization uptake was evaluated using the WIR, an internet database tracking immunization dates of Wisconsin children and adults since 2000, available within the EHR.^12^ Immunizations provided by both public and private providers in Wisconsin are uploaded into the registry, and 98.5% of Wisconsin adults have an active WIR.^13^ Studies have demonstrated that the WIR captures 97% of vaccines administered in Wisconsin.^14^ The WIR has been used to evaluate influenza and COVID-19 vaccine uptake among patients with IBD.^4,7^

### Outcomes

Our primary outcome was influenza immunization rates among patients with IBD for the 2018–2019 and 2019–2020 influenza seasons. We chose both seasons because an annual influenza vaccine is recommended.^15^ Our secondary outcomes included pneumococcal, herpes zoster, and hepatitis B immunization rates, all vaccines recommended for adult patients with IBD. Pneumococcal immunization was evaluated only in patients with IBD receiving immunosuppressive therapy and was defined as the administration of the 13-valent pneumococcal conjugate vaccine (PCV13) and 23-valent polysaccharide vaccine (PPSV23). We also collected pneumococcal immunization status for those aged 65 years and older who had received a PPSV23. Herpes zoster immunization was defined as the completion of a recombinant zoster vaccine two-dose series in patients with IBD who were aged 50 years and older. Hepatitis B immunization was defined as completing any vaccine series or having a hepatitis B surface antibody level greater than 10 mIU/ml and no other hepatitis B immune markers.

### Statistical Analyses

We summarized demographic information using descriptive statistics of raw counts and percents for categorical data and mean plus standard deviation for continuous data. All categorical data were statistically compared using chi-squared tests. We compared numerical data using the analysis of variance (ANOVA) for all normally distributed variables. Non-normal data were compared using Kruskal–Wallis tests. We conducted a univariate regression analysis and a multivariate model adjusting for factors from the univariate analysis. We used a Poisson regression with robust standard errors to compute prevalence ratios with 95% confidence intervals and *P*-values. Variables were checked for multicollinearity in the multivariate models using a variance inflation factor (VIF). All VIF values were less than 10 and therefore did not warrant further action or corrections. Significant *P*-values were those ≤ 0.05. We conducted all analyses using STATA version 17.

### Ethics

This study was approved by the Institutional Review Boards at University of Wisconsin-Madison and Medical College of Wisconsin.

## Results

**Figure 1.**
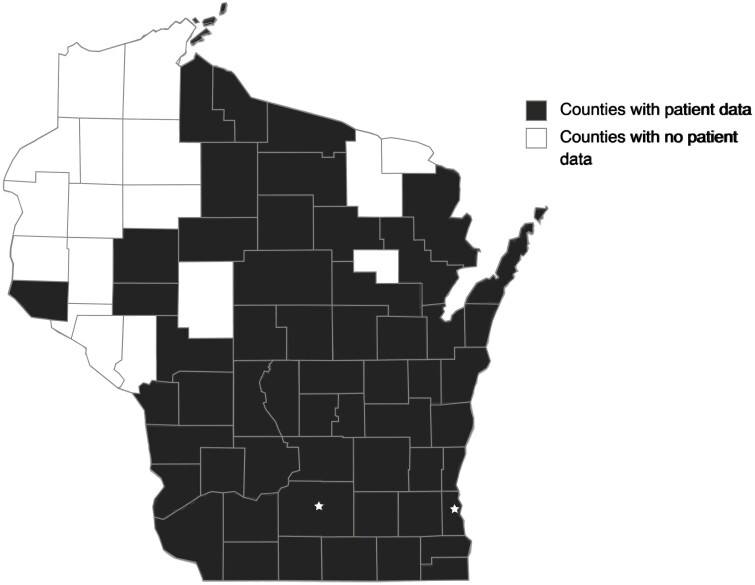
Wisconsin county map. Participants reside in the shaded counties.

We identified 2453 patients with IBD who met the inclusion criteria from both institutions (Table 1). Most patients self-identified racially/ethnically as non-Hispanic White (*n* = 2205). Most patients were on immunosuppressive therapy (74.5%) and lived in urban areas (80.1%), and many lived in socioeconomically advantaged areas (48.7%) (Tables 1 and 2). Patients represented 55 of 72 counties in Wisconsin, including the most racial/ethnically diverse counties (Figure 1). The majority (99/135, 73%) of black patients lived in urban disadvantage zip codes. Almost half (19/45 42%) of Hispanic patients lived in a rural or urban disadvantage zip code.

**Table 1.** Demographics table for race or ethnicity.

Race/ethnicity										
		American Indian/Alaska Native	Asian	Black	Native Hawaiian/other Pacific Islander	Non-Hispanic White	Other	Unknown	All Hispanic/ Latino	*P-*value
N		8	37	135	4	2205	11	6	45	
Mean age (years)		33.9 ± 15.2	41.9 ± 16.1	43.2 ± 15.3	29.5 ± 6.6	47.2 ± 17.4	42.1 ± 16.1	44.1 ± 14.9	41.4 ± 18.9	.001
Sex	Male	3 (37.5%)	19 (51.4%)	65 (48.2%)	0 (0.0%)	1024 (46.4%)	6 (54.5%)	3 (50.0%)	18 (40.0%)	.62
BMI		28.0 ± 5.7	25.3 ± 5.9	29.8 ± 8.0	25.5 ± 3.7	28.0 ± 6.5	26.3 ± 6.0	27.8 ± 5.7	25.5 ± 5.7	.001
Diagnosis	Crohn’s disease	5 (62.5%)	16 (43.2%)	98 (72.6%)	1 (25.0%)	1371 (62.2%)	5 (45.5%)	3 (50.0%)	19 (42.2%)	
	Ulcerative Colitis	3 (37.5%)	21 (56.8%)	37 (27.4%)	3 (75.0%)	828 (37.6%)	6 (54.5%)	3 (50.0%)	26 (57.8%)	.043
	Other	0 (0.0%)	0 (0.0%)	0 (0.0%)	0 (0.0%)	6 (0.2%)	0 (0.0%)	0 (0.0%)	0 (0.0%)	
Mean IBD duration (years)		10.3 ± 9.8	9.4 ± 10.1	12.9 ± 9.9	9.2 ± 6.8	15.1 ± 12.6	16.2 ± 8.4	20.6 ± 16.1	12.2 ± 12.4	.016
IBD treatment	5-ASA	4	15	22	1	578	3	2	19	
	AZA or monotherapy	0	5	10	0	168	0	0	1	
	MTX monotherapy	0	0	2	0	24	0	0	0	
	anti-TNF monotherapy	3	7	50	1	609	2	3	10	
	anti-TNF + AZA/6-MP	0	3	12	0	185	1	0	3	
	anti-TNF + MTX	0	1	2	0	42	0	1	1	
	Vedolizumab monotherapy	0	4	12	1	246	2	0	7	
	Vedolizumab + AZA/6-MP	0	0	1	0	30	0	0	0	
	Vedolizumab + MTX	0	0	0	0	8	0	0	0	
	Ustekinumab	0	2	15	0	213	2	0	3	
	Ustekinumab + AZA/6-MP	0	1	3	0	29	0	0	2	
	Ustekinumab + MTX	0	0	1	0	12	0	0	0	
	Tofacitinib	0	1	0	0	44	0	0	1	
	Corticosteroid	3	7	16	1	378	2	2	7	
Immunosuppressed^*^	Yes	5 (62.5%)	24 (64.9%)	98 (72.65)	3 (75.0%)	1656 (75.1%)	7 (63.6%)	5 (83.3%)	29 (64.4%)	.49

Abbreviations: 5-ASA: 5-aminosalicylic acid; 6-MP: 6-mercaptopurine; AZA: azathioprine; anti-TNF: anti-tumor necrosis factor; MTX: methotrexate.

^*^Immunosuppressed includes those on azathioprine, methotrexate, anti-TNF, ustekinumab, tofacitinib, and/or corticosteroid.

**Table 2.** Demographics table for ZIP code tabulation areas.

		Urban—underserved	Urban	Urban—advantaged	Rural—underserved	Rural	Rural—advantaged	Other ^*^	*P*-value
*N*		209	699	970	29	265	172	101	
Mean age (years)		43.3 ± 16.8	46.7 ± 17.5	46.6 ± 17.6	52.2 ± 15.8	49.8 ± 16.8	48.7 ± 16.1	43.5 ± 17.9	<.001
Sex	Male	100 (47.8%)	336 (48.1%)	447 (46.1%)	14 (48.3%)	119 (44.9%)	74 (43.0%)	46 (45.5%)	.911
BMI		28.6 ± 7.5	28.1 ± 6.5	27.3 ± 6.2	32.6 ± 6.2	29.0 ± 7.5	28.7 ± 5.8	27.1 ± 6.6	<.001
Diagnosis	Crohn’s disease	141 (67.5%)	467 (66.8%)	549 (56.6%)	19 (65.5%)	156 (58.9%)	114 (66.3%)	68 (67.3%)	
	Ulcerative colitis	68 (32.5%)	230 (32.9%)	419 (43.2%)	10 (34.5%)	109 (41.1%)	56 (32.6%)	33 (32.7%)	.001
	Other	0 (0.0%)	2 (0.3%)	2 (0.2%)	0 (0.0%)	0 (0.0%)	2 (1.2%)	0 (0.0%)	
Mean IBD duration (years)		13.5 ± 11.1	16.4 ± 12.9	14.5 ± 12.6	12.8 ± 12.5	13.9 ± 12.1	16.3 ± 13.0	13.0 ± 10.8	.002
IBD treatment	5-ASA	39	126	328	8	92	34	15	
	AZA or monotherapy	13	47	79	5	19	12	8	
	MTX monotherapy	4	6	9	0	4	1	2	
	anti-TNF monotherapy	70	199	247	6	77	51	32	
	anti-TNF + AZA/6-MP	23	70	65	2	22	14	8	
	anti-TNF + MTX	4	12	22	0	6	2	1	
	Vedolizumab monotherapy	22	95	101	3	25	17	9	
	Vedolizumab + AZA/6-MP	2	9	10	0	6	2	0	
	Vedolizumab + MTX	0	3	2	0	2	1	0	
	Ustekinumab	27	80	70	4	19	22	13	
	Ustekinumab + AZA/6-MP	4	17	11	0	1	0	2	
	Ustekinumab + MTX	1	3	6	1	0	1	1	
	Tofacitinib	1	15	21	0	3	4	3	
	Corticosteroid	25	98	181	7	56	27	22	
Immunosuppressed	Yes	157 (75.1%)	546 (78.1%)	681 (70.2%)	24 (82.8%)	196 (74.0%)	132 (76.7%)	86 (85.1%)	.001

Abbreviations: 5-ASA: 5-aminosalicylic acid; 6-MP: 6-mercaptopurine; AZA: azathioprine; anti-TNF: anti-tumor necrosis factor; MTX: methotrexate.

^*^Other includes those ZIP codes that had only PO boxes, commercial or organization ZIP codes, or populations less than 500.

ZIP code tabulation areas are derived from Wisconsin Collaborative for Healthcare Quality and the University of Wisconsin Health Innovation Program. Wisconsin Health Disparities Report: Rural and Urban Populations, 2020. https://www.wchq.org/disparities.

The Wisconsin Zip code tabulation areas and their corresponding categories can be downloaded at https://www.hipxchange.org/RuralUrbanGroups.

### Influenza Vaccine Uptake

**Figure 2.**
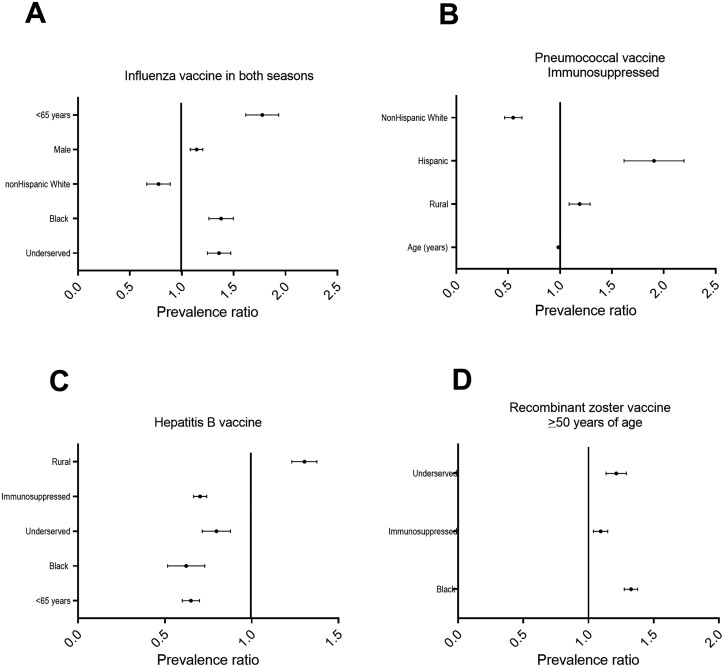
Prevalence ratios (PR) for factors for receiving adult vaccines. A. Influenza vaccine in both 2017–2018 and 2018–19 seasons. Older age (PR 0.98; 95% confidence interval (95%CI) 0.98–0.99; *P* < .001), and non-Hispanic White patients (PR 0.76, 95% CI 0.59–0.98, *P* < .03) were significantly more likely to receive the influenza vaccine in both seasons. Male patients (PR 1.14; 95%CI 1.04–1.25; *P* = .004), Black patients (PR 1.37; 95%CI 1.18–1.59; *P* < 0.001) and those living in underserved geographic areas (PR 1.35; 95%CI 1.17–1.56; *P* < 0.001) were less likely to be immunized against influenza in both seasons. B. Pneumococcal vaccines (both pneumococcal conjugate 13-valent and pneumococcal polysaccharide 23-valent vaccines as recommended for immunosuppressed patients). Younger age PR 0.98; (95%CI 0.98–0.99; *P* < .001), and those who were not non-Hispanic White patients (PR 0.54; 95% CI 0.41–0.70; *P* < .001) were significantly more likely to have not completed the pneumococcal vaccine series. Hispanic patients were less likely to have completed the series compared to all others (PR 1.86; 95% CI 1.42–2.43; *P* < .001). Those living in rural areas were less likely to have completed the series (PR 1.18; 95% CI 1.02–1.37; *P* = .03). C. Hepatitis B vaccine. Unlike other vaccines, Black patients (PR 0.60; 95%CI 0.45–0.80; *P* < 0.001), patients on immunosuppressive therapy (PR 0.70; 95%CI 0.64–0.77; *P* < .001) and those living in an underserved area (PR 0.79; 95%CI 0.66–0.94; *P* = .009) were all associated with hepatitis B vaccine uptake. Patients who were older in age (PR 1.02; 95%CI 1.02–1.02; *P* < .001), non-Hispanic White (PR 1.19; 95%CI 1.00–1.40; *P* = .05) and rural-dwelling (PR 1.30; 95%CI 1.18–1.43; *P* < .001) were associated with less likely to have completed the hepatitis B vaccine series. D. Recombinant zoster vaccine for those aged 50 years and older. Black patients (PR 1.32; 95% 1.24–1.42; *P* < .001), patients on immunosuppressive therapy (PR 1.09; 95% CI 1.00–1.19; *P* = .05) and those living in an underserved area (PR 1.21; 95%CI 1.08–1.35; *P* = 0.001) were less likely to have received the recombinant zoster vaccine series.

The majority of patients (1374/2453; 56.0%) received influenza vaccines during the 2018–2019 and 2019–2020 seasons. Older age (prevalence ratio (PR) 0.98; 95% confidence interval (95%CI) 0.98–0.99; *P* < .001) and non-Hispanic White patients (PR 0.76, 95% CI 0.59–0.98, *P* < .03) were significantly more likely to receive the influenza vaccine in both seasons. Male patients (PR 1.14; 95%CI 1.04–1.25; *P* = .004), Black patients (PR 1.37; 95%CI 1.18–1.59; *P* < .001), and those living in underserved geographic areas (PR 1.35; 95%CI 1.17–1.56; *P* < .001) were less likely to be immunized against influenza in both seasons (Figure 2A). In the multivariable analysis, older age and non-Hispanic white patients were more likely to have to receive the influenza vaccine, while patients identifying as male were less likely. The remaining variables (Hispanic, geographic location, being on immunosuppressive therapy) were not statistically significant contributors to the model (Table 3).

**Table 3.** Multivariable models for prevalence ratio not receiving the vaccine influenza in both seasons.

No *n* = 1079; yes *n* = 1374						
	Univariable			Multivariable		
	PR	95% CI	*P*	PR	95% CI	*P*
Age (continuous variable)	0.98	0.98–0.99	<.001*	0.99	0.98–0.99	<.001*
<65 years	1.76	1.51–2.06	<.001*			
Male	1.14	1.04–1.25	.004*	1.16	1.01–1.34	.03*
Hispanic	1.29	0.99–1.66	.05*			
Non-Hispanic White	0.76	0.59–0.98	.03*	0.71	0.52–0.97	.03*
Black	1.37	1.18–1.59	<.001*	1.23	0.89–1.71	.21
Rural	1.04	0.93–1.16	.48			
On immunosuppressive therapy	1.10	0.99–1.23	.07			
Underserved	1.35	1.17–1.56	<.001*	1.10	0.68–1.36	.82
Both pneumococcal vaccines for those younger than age 65 years and immunosuppressed						
No *n* = 578; yes *n* = 949						
Age (continuous variable)	0.98	0.98–0.99	<.001*	0.99	0.98–0.99	<.001*
Male	1.02	0.89–1.15	.82			
Hispanic	1.86	1.43–2.43	<.001*	1.68	1.27–2.22	<.001*
Non-Hispanic White	0.54	0.41–0.70	<.001*			
Black	0.89	0.67–1.20	.46			
Rural	1.18	1.02–1.37	.03*	1.24	1.07–1.45	.004*
Underserved	0.97	0.76–1.23	.77			
**Hepatitis B vaccine series**						
No, *N* = 1073; Yes, *N* = 1380						
Age (continuous variable)	1.02	1.02–1.02	<.001*	1.02	1.02–1.02	<.001*
<65 years	0.66	0.61–0.73	<.001*			
Male	1.00	0.92–1.10	.98			
Hispanic	1.08	0.81–1.48	.54			
Non-Hispanic White	1.19	1.00–1.40	.05*	0.76	0.61–0.93	.009*
Black	0.60	0.45–0.80	<.001*	0.50	0.34–0.74	.001*
Rural	1.30	1.18–1.43	<.001*	1.18	1.02–1.35	.02*
On Immunosuppressive therapy	0.70	0.64–0.77	<.001*	0.79	0.71–0.89	<.001*
Underserved	0.79	0.66–0.94	.009*	0.95	0.79–1.14	.56
**Zoster vaccine for those age ≥50 years**						
No, *n* = 746; yes, *n* = 268						
Age (continuous variable)	0.99	0.99–1.00	.13			
Male	0.94	0.88–1.02	.14			
Hispanic	1.18	0.97–1.45	.11			
Non-Hispanic White	0.83	0.68–1.02	.08			
Black	1.32	1.24–1.42	<.001*			
Rural	0.98	0.89–1.07	.62			
On immunosuppressive therapy	1.09	1.00–1.19	.05*	1.07	0.95–1.20	.25
Underserved	1.21	1.08–1.35	.001*	1.09	0.93–1.27	.30

### Pneumococcal Vaccine Uptake

Among the immunosuppressed patients younger than age 65 years in this cohort, 949/1527 (62.1%) received both PCV13 and PPSV23. Older age (PR 0.98; 95%CI 0.98–0.99; *P* < .001) and non-Hispanic White patients (PR 0.54; 95% CI 0.41–0.70; *P* < .001) were significantly more likely to have completed the pneumococcal vaccine series. Hispanic patients were less likely to have completed the series compared to all others (PR 1.86; 95% CI 1.42–2.43; *P* < .001). Those living in rural areas were less likely to have completed the series (PR 1.18 (95% CI 1.02–1.37; *P* = .03) (Figure 2B). No difference was identified in completing the pneumococcal vaccine series for those dwelling in underserved compared to advantaged areas (PR 0.97; 95%CI 0.76–1.53; *P* = .77). In the multivariable analysis, only older age was associated with being more likely to have completed the series while Hispanic patients and those living in rural areas were less likely to have completed the pneumococcal vaccine series. The remaining variables were not statistically significant contributors to the model.

For those aged 65 years and over, the rate for any pneumococcal vaccine was 417/435 (95.9%). In univariate analysis, none of the independent variables (age, identifying as male, race, ethnicity, geographic location, or being on immunosuppressive therapy) were associated with vaccine uptake (data not shown).

### Hepatitis B Vaccine Uptake

Among our study population, 1380 of 2453 patients (56.3%) completed a hepatitis B vaccine series. In the univariate analysis, Black patients (PR 0.60; 95%CI 0.45–0.80; *P* < .001), patients on immunosuppressive therapy (PR 0.70; 95%CI 0.64–0.77; *P* < .001), and those living in an underserved area (PR 0.79; 95%CI 0.66–0.94; *P* = .009) had higher hepatitis B vaccine uptake. Patients who were older (PR 1.02; 95%CI 1.02–1.02; *P* < .001), non-Hispanic White (PR 1.19; 95%CI 1.00–1.40; *P* = .05) and those living in rural areas (PR 1.30; 95%CI 1.18–1.43; *P* < .001) were less likely to have completed the hepatitis B vaccine series (Figure 2C). In the multivariable model, Black patients and those on immunosuppressive therapy were more likely to have completed a hepatitis B vaccine series, while those of older age and non-Hispanic White patients were still less likely to have completed the series. The remaining variables were not statistically significant contributors to the model (Table 3).

### Herpes Zoster Vaccine Uptake

The recombinant zoster immunization rate in our cohort of patients aged 50 years and older was 268/1011 (24.3%). In the univariate analysis, Black patients (PR 1.32; 95% 1.24–1.42; *P* < .001, patients on immunosuppressive therapy (PR 1.09; 95% CI 1.00–1.19; *P* = .05), and those living in an underserved area (PR 1.21; 95% CI 1.08–1.35; *P* = .001) were less likely to have received the recombinant zoster vaccine series (Figure 2D). In the multivariable model, Black patients were less likely to have completed the series; this was the only significant variable in the model (Table 3). The remaining variables were not statistically significant contributors to the model.

## Discussion

In this multicenter study, we found that racial, ethnic, and geographic disparities exist among adult patients with IBD. Non-Hispanic White patients were more likely to receive an influenza vaccine or have completed a pneumococcal vaccine series compared to other races and ethnicities. Hispanic patients were less likely to have completed the pneumococcal vaccine series compared to other groups. We also found that Black patients eligible to be vaccinated against zoster were less likely to have completed the zoster vaccine series. Disparities in these three vaccines are clinically significant since they are the three most common VPDs resulting in a serious infection.^16^ In contrast, to these three VPDs, we did not see disparities in hepatitis B vaccine uptake where Black patients were more likely to have been immunized compared to non-Hispanic Whites.

Similarly, our use of a geodisparity model, allowed us to unmask disparities in geographically underserved areas compared to geographically advantaged areas when there was no difference in overall rural compared to urban. Those in underserved areas were less likely to have received influenza vaccines in both seasons, completed a pneumococcal vaccine series, or received the zoster vaccine series compared to those in areas of advantage. Uptake in the pneumococcal vaccines series was the only disparity seen when comparing people living in rural vs. urban areas. Similarly, we found a contrasting finding in hepatitis B vaccine uptake, those in underserved areas were more likely to get vaccinated compared to those in an advantaged area while those in rural areas where less likely to have been vaccinated for hepatitis B compared to those in urban areas.

Hepatitis B infection may occur at higher rates in urban areas and among Asian people and non-Hispanic Black people.^17^ Up until very recently, hepatitis B vaccine recommendations targeted individuals at higher risk for infection.^18^ Nearly 90% of Black people in Wisconsin live in five urban counties and one county with urban and rural areas.^10,11,19^ The patterns identified in this study were consistent with the former Advisory Committee on Immunization Practices recommendations for hepatitis B vaccine use in adults which were primarily based on risk for infection. Importantly, hepatitis B vaccine series is the only vaccine in this study that is included in the childhood immunization recommendations. Routine vaccination of infants with a three-dose hepatitis B series has been recommended since 1991 by the Advisory Committee on Immunization Practices (ACIP) in the United States to target hepatitis B for elimination. In 1997, recommendations expanded to include vaccination of any children aged 0–18 years who had not yet been vaccinated.^20,21^ Childhood immunization rates are much higher than adult rates and were bolstered by the introduction of the vaccines for Children program which provides vaccines at no cost to uninsured and underinsured children.^22^ These results highlight racial and ethnicity disparities that might be addressed by eliminating vaccine costs and improving access and also show that geographic disparities exist beyond the rural–urban divide, and that not all rural and all urban communities share the same healthcare experience.

Disparity research in the IBD population has historically neglected geographic disparities. Geographic disparities due to inequities in social determinants of health may be missed if focusing on racial and ethnic disparities alone. Health care access and transportation are important elements of social determinants of health that disproportionately impact the care of certain patients with IBD. Patients with IBD who live in rural areas have fewer visits to IBD specialists, a higher number of emergency department visits, and Crohn’s disease-related hospitalizations compared to urban IBD patients.^23^ In the general population, rural minority populations have worse health outcomes and access to healthcare compared to non-Hispanic White people.^24^ Future disparity research in IBD should also focus on geographic disparities to identify ways to improve care.

The cause of lower immunization rates in medically underserved populations is complex and multifactorial, including limited access to care, lack of or suboptimal health insurance coverage, and structural racism.^25,26^ These elements ultimately lead to fewer ambulatory care visits and missed opportunities for preventative health care, including fewer chances to be immunized.^27^ A recent study found that patients who lived in rural areas had fewer outpatient visits with their gastroenterologist in addition to higher rates of IBD-related hospitalizations and emergency department visits compared with those living in urban areas.^23^ Similarly, racial and ethnic disparities exist regarding access to gastroenterology visits.^28,29^

Additional contributors to lower vaccine uptake involve patient-specific and systemic factors. Patient hesitancies include concerns about vaccination side effects, lack of knowledge regarding the recommendations for vaccines, and misconceptions about vaccinations.^30^ Systemic barriers include clinic vaccine supply, provider recommendations, and workflow amenable to vaccine administration. A survey of 75 IBD centers across the United States found that 36% of respondents could not administer vaccines due to cost of stocking vaccines in clinics, inadequate storage, lack of staff to administer vaccines, or reimbursement concerns.^31^ Successful quality improvement efforts to improve vaccination rates in patients with IBD have included administering a vaccine questionnaire to patients in clinic and dedicating a nurse to communicate vaccine requirements to primary care providers.^32^ Finally, gastroenterology providers should consider offering vaccines in clinics during routine appointments or using local pharmacies if providing vaccines in clinics is not possible to improve access to care.^33,34^

Overcoming barriers to immunization for patients with IBD requires a multi-faceted approach including better access to subspecialty care, improved health insurance coverage through Medicaid expansion, and a more diverse health care workforce.^35,36^ Gastroenterology providers should share the responsibility with primary care providers in assuring their patients are up to date with all required vaccines.^37^ It is critical that patients receive objective, non-biased information regarding the risks of VPDs and the benefits of vaccinations so they can make an informed decision.

Our study has several strengths that make our findings generalizable to other centers. We were able to verify vaccine uptake using a statewide immunization registry whereas other vaccine coverage studies often rely on participant survey responses.^38^ We evaluated a large cohort of patients with IBD prior to the COVID-19 pandemic when access to care or telemedicine may have impacted adult vaccine uptake. We used ZIP codes to report urban versus rural data and included a wide demographic of patients. Our study also adds a new, important dimension to IBD disparity research by including urban versus rural data with advantaged and underserved classifications, alongside racial and ethnic data, allowing us to reveal disparities that would have otherwise been missed. Underserved areas have access to fewer healthcare providers, experience higher rates of poverty, have a greater proportion of uninsured and Medicaid patients, have lower education attainment, and have overall poorer health status at a population level in comparison to advantaged ZIP codes.^11^

There were several limitations to our study. The state of Wisconsin lacks diversity compared to the overall United States, with a diversity index similar to only nine other states.^39^ In our cohort of patients, Milwaukee County, Wisconsin’s most racially and ethnically diverse urban county, had disparities similar to the rest of the state. Therefore, we did not further analyze county data. Because our population under-represents racial and ethnic minorities, the vaccination disparities in other states may differ. Race and ethnicity were defined within the social constructs of the EHR, and some racial/ethnic groups had relatively small numbers, thus they were combined for most analyses. These factors unintentionally imply a generalized experience and may mask unique differences among various racial and ethnic groups. Other limitations included: a lack of documentation of whether patients were offered vaccines, reasons for incomplete vaccination, and short study duration. Additionally, our study population likely over-represented patients with adequate health insurance coverage, as being seen in a gastroenterology clinic was among the inclusion criteria.

## Conclusion

Our study showed that patients with IBD from traditionally medically underserved populations have lower vaccine uptake. The demographics of IBD are rapidly changing in the United States, where racial and ethnic minorities are increasingly being diagnosed with IBD. Vaccine uptake is a vital part of IBD care, and it is imperative to identify barriers and implement improvement strategies to address health inequalities and close this gap.

## Data Availability

The data may be available please contact corresponding author for any request.
